# Criteria adherence and citation impact of urologic Cochrane review co‐publications

**DOI:** 10.1002/cesm.12004

**Published:** 2023-03-27

**Authors:** Ranveer Vasdev, Zahrah Shakur, Philipp Dahm

**Affiliations:** ^1^ School of Medicine University of Minnesota Twin Cities Minneapolis Minnesota USA; ^2^ Cochrane Urology Group Minneapolis Minnesota USA; ^3^ Urology Section Minneapolis Veterans Affairs Health Care System Minneapolis Minnesota USA

**Keywords:** Cochrane reviews, co‐publication, evidence based medicine, urology

## Abstract

**Introduction:**

Cochrane systematic reviews are widely recognized as authoritative sources of evidence. To improve dissemination and impact, editorial groups often encourage co‐publication of their reviews in other journals. Our study aimed to analyze urology‐relevant co‐publications and determine their adherence to Cochrane's four co‐publication criteria and impact using citation analysis

**Methods:**

We systematically identified all Cochrane reviews published by the Urology, Incontinence, Renal, and Transplantation Groups from 1998 to 2021 as well as subsequent co‐publications using MEDLINE, Web of Science, Scopus, and Google Scholar databases. We also determined adherence to Cochrane's four co‐publication criteria and analyzed citation rates.

**Results:**

Of the 202 Cochrane reviews included, 52 (25.7%) had an associated co‐publication. The majority of the co‐publications corresponded to the Urology Group (39; 76.9%), followed by the Incontinence (9; 17.3%) and Kidney and Transplant Group (3; 5.8%). Only 21 (40.0%) co‐publications met all four co‐publication criteria, with the most common criteria not satisfied was inclusion of the word “Cochrane” in the co‐publication title (50% adherence). The proportion adhering to a subset of criteria significantly increased for reviews published between 2013 and 2021 compared to those from 1998 to 2012. Compared to corresponding Cochrane reviews, there was no significant difference in the number of citations of co‐publications across all sampled databases, although co‐publication citations were usually less than those of original reviews.

**Conclusion:**

Approximately one in four urology‐related Cochrane reviews are co‐published. Though co‐publications garnered a considerable number of citations that could help in the dissemination of Cochrane reviews, many are not readily identifiable as such.

## 1 INTRODUCTION

Cochrane is widely recognized as a not‐for‐profit organization committed to the development and dissemination of systematic reviews which are published in the Cochrane Database of Systematic Reviews (CDSR). [1] To promote transparency and reproducibility, a single Cochrane review will frequently have hundreds of printed pages. However, this length is difficult to digest for most clinical readers, who may also not be aware of the study in the first place due to its exclusive location in the CDSR. Therefore, to enhance the dissemination of these reviews to a broader audience, Cochrane has, since its beginnings, allowed co‐publication in specialist journals as long as co‐publications met certain criteria, including that the co‐publication met the same quality standards as the original and also explicitly referenced the original Cochrane review as its source. [2] Co‐publications of Cochrane reviews are not duplicate publications or academic misconduct, but a way to improve access to Cochrane evidence to the readers of specialists’ journals in a much abbreviated, and oftentimes more appealing format than the original review.

A recent study assessing the quality of systematic reviews in the urological literature from 2016 to 2018 included several co‐published Cochrane reviews which were of high methodological quality. [3] However, there is no study to date that has broadly investigated co‐publication practices across all urologic Cochrane systematic reviews. Thus, the aim of our study is to formally assess the practice of co‐publication for urology Cochrane reviews by determining adherence to Cochrane's four co‐publication criteria and measuring scientific impact via citation analysis.

## 2 MATERIALS AND METHODS

### 2.1 Data source

We systematically identified all reviews published from the year of the first urologic Cochrane review publication (1998) up until December 28, 2021 by the Urology, Incontinence, and Renal and Transplantation Cochrane Editorial Groups. The latter two editorial groups were included as they often included many urology‐related reviews. As such Renal and Transplant reviews were included if they concerned urologic topics, as listed in Supporting Information: Appendix 1. Additionally, Incontinence reviews concerning fecal incontinence were omitted and those concerning urinary incontinence were included. Three Incontinence Group reviews concerning surgical management of pelvic organ prolapse were originally published by this Group however, later updates were published in the Gynecology and Fertility Editorial Group. As surgical management of this condition is often performed by urologists and urogynecologists and as these reviews are listed under the “Our Reviews” section of the Cochrane Incontinence Group website, they were included in our study. The following features for each review article were collected: urologic area of study, authors, corresponding author institution, journal name, number of review updates, and the publication date of the original and most updated review. The number of review citations was determined using MEDLINE, Web of Science, Scopus, and Google Scholar databases.

The goal of Cochrane co‐publications is to improve the dissemination of systematic reviews via publication of abbreviated review findings in specialty journals or languages other than English. The application process of co‐publication typically occurs postpublication of the original review and requires approval by the Cochrane Review Group, Cochrane Editorial & Methods Department, and the editorial team of intend co‐publication journal. Several journals have pre‐existing co‐publication agreements with Cochrane, including BJU International, Canadian Urological Association Journal, and Investigative and Clinical Urology. Additional details on the process of co‐publication agreement process can be found online. [2]

Screening for co‐publications was first performed using the MEDLINE single citation matcher with the first author's full name and a keyword from the review's title. We then looked at studies under the “Cited by” section on MEDLINE. If no co‐publications were identified, we then searched Web of Science, Scopus, and Google Scholar databases using the title of the review AND the first author's surname OR last author's surname as search criteria. If no co‐publications were found in these four databases, we concluded the review was not associated with a co‐publication. Disagreement on co‐publication status was resolved via discussion between authors (Ranveer Vasdev and Zahrah Shakur). The screening was performed in English only.

For all co‐publications, the following data were collected: authorship, primary author institution, and publication journal. Then using MEDLINE, Web of Science, Scopus, and Google Scholar databases we recorded the total number of times each co‐publication article was cited. There are a total of five Cochrane's co‐publication criteria however, one, where co‐publication authors must notify journal editors that their submission is based on a Cochrane Review, could not be assessed externally. Thus of the four remaining co‐publication criteria, two authors (Ranveer Vasdev and Zahrah Shakur) determined adherence to said criteria as defined as the following: (1) the co‐publication faithfully reflects the data and interpretation of the Cochrane version, (2) the title of the co‐publication indicates that it is a secondary publication of the Cochrane review, (3) the co‐publication acknowledges the Cochrane review group in an appropriate place, and (4) the co‐publication cities the Cochrane review in its reference list. [2] Interobserver differences in adherence criteria was assessed using a subsample of co‐publications that were cross evaluated by two authors (Ranveer Vasdev and Zahrah Shakur). Adequate interobserver agreement was determined apriori as agreement greater than 80% per each co‐publication criterion.

### 2.2 Outcome definition

The primary outcomes were the number of citations of co‐publications compared to corresponding reviews across all sampled and adherence to co‐publication criteria.

### 2.3 Statistical analysis

Summary statistics were gathered for all reviews and all co‐publications. Bivariate analyses were performed to compare the number of database‐specific citations between reviews and associated co‐publications. Association analysis of co‐publication criteria adherence was performed. Additionally, we compared adherence rates of publications published between 1998 and 2012 versus 2013 and 2022. These specific ranges were selected based on the distribution as they divided co‐publications into near‐equal halves. Chi‐squared and Wilcoxon Rank Sum Tests were used as appropriate. A *p*‐value of 0.05 was used as criteria for statistical significance. Review and co‐publication data were stored using Microsoft Excel (v16.60) and analyses were performed using R (v4.1.1).

## 3 RESULTS

Of the 421 Cochrane reviews published by three editorial groups, 202 reviews published between 1998 and 2021 met inclusion criteria (Supporting Information: Appendix 2). Most reviews were published by the Urology Editorial Group (*n* = 87; 43.1%), followed by the Incontinence (*n* = 79; 39.1%) and Kidney and Transplant (*n* = 36; 17.8%) Groups. The most common review topics were female incontinence and pelvic reconstruction (61; 30.2%), oncology (49; 24.2%), and infection/inflammation (34; 16.8%).

Of the 202 reviews, 52 (25.7%) had an associated co‐publication. The majority of the co‐published reviews corresponded to the Urology Group (39; 76.9%), followed by the Incontinence (9; 17.3%) and Kidney and Transplant Groups (3; 5.8%). These co‐publications were distributed across 18 different journals of which most were urology specialty journals (39; 75.0%). The three leading journals of co‐publication were BJU International (21; 40.4%), followed by Neurourology and Urodynamics (6; 11.5%) and European Urology (4; 7.7%). We identified one Cochrane review which has two associated co‐publications. The first co‐publication was associated with the original review while the second co‐publication was associated with an updated review. Both co‐publications were included for analysis. To the best of our knowledge, there were no co‐publication updates of previous co‐published reviews.

**Figure 1:**
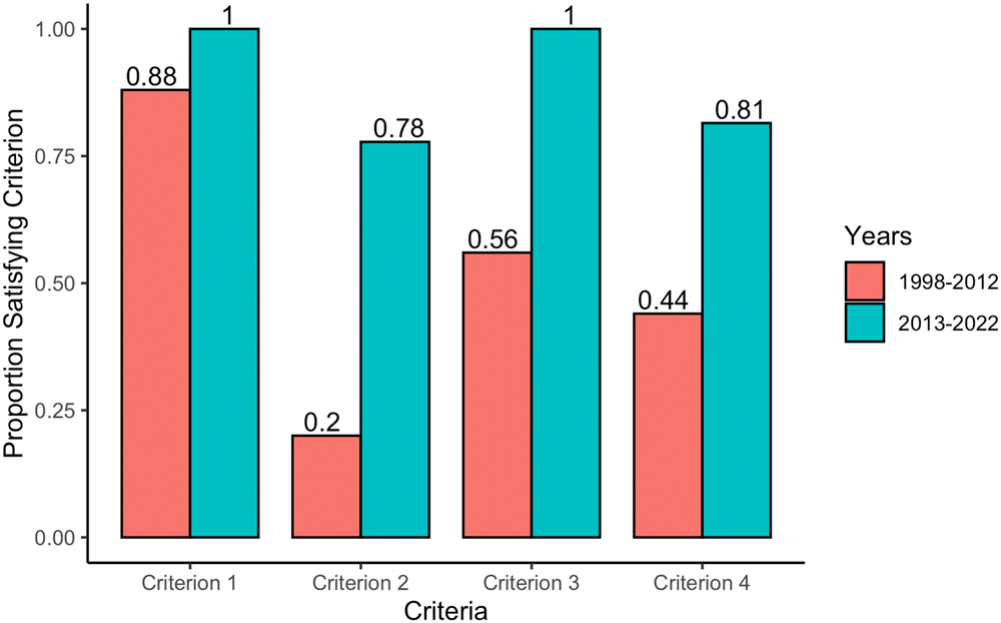
Proportion of co‐publications satisfying individual criterion Criteria 1: the co‐publication faithfully reflects the data and interpretation of the Cochrane version. Criteria 2: the title of the co‐publication indicates that it is a secondary publication of the Cochrane review. Criteria 3: the co‐publication acknowledges the Cochrane review group in an appropriate place. Criteria 4: the co‐publication cities the Cochrane review in its reference list [2].

Overall, only 21 (40.0%) co‐publications met all four co‐publication criteria. The criterion mandating that the described review have the exact same results as the underlying Cochrane review (criterion #1) was met by most co‐publications (49; 94.2%), followed by explicit acknowledgment of the corresponding review in the co‐publication text (criterion #3: 41; 78.9%). Approximately two‐thirds of co‐publications also cited the source review (criterion #4; 34; 63.4%), but less than half included “Cochrane” in its title (criterion #2; 26; 50.0%). When comparing co‐publications from the earlier (1998 to 2012) and later (2013–2022) time‐periods, reporting, the proportion (number) adhering to Criteria 2, 3, and 4 significantly improved (Criteria 2, 20% [5] vs. 77% [21], *p* < 0.001; Criteria 3, 56% [14] vs. 100% [27], *p* < 0.001; Criteria 4, 44% [11] vs. 81% [22], *p* = 0.009) (Figure 1).

**Figure 2:**
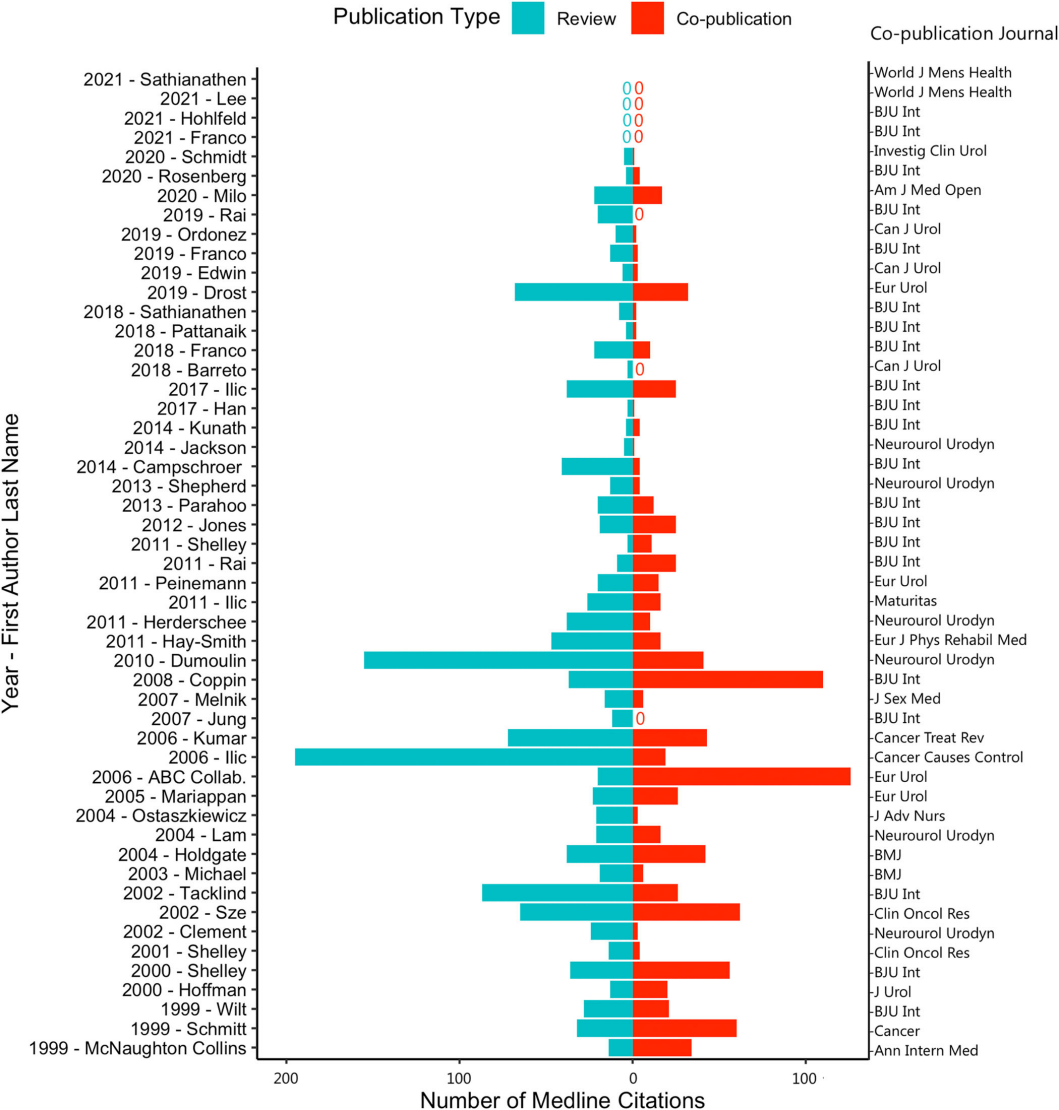
Citations of co‐publications and reviews by author, year, and co‐publication journal. Co‐publication journal names were abbreviated according to the National Library of Medicine Catalogue. Two co‐publications were associated with the same series of Cochrane review (one with the original review and one with the updated review). As we are comparing the number of citations of co‐publications compared to citations of any review (original or updated), the co‐publication associated with the updated review was excluded from this figure to allow for comparisons for all other co‐publication and review pairs.

The median (interquartile range) number of citations for the original Cochrane reviews based on MEDLINE, Web of Science, Scopus, and Google Scholar was 16 (7–32), 24 (9–54), 44 (9–98), and 90 (31–181), respectively. When limiting the analysis to those reviews that were co‐published, the median (interquartile range) number of citations for original reviews was greater than that of co‐publications across all databases, except for the Web of Science database. However, these differences were not statistically significant {MEDLINE (19.5 [5.25–35] vs. 11 [2–26]), Web of Science (31 [8.25–53.5] vs. 34 [3.0–74]), Scopus (44.5 [11–97.5] vs. 39 [4.0–76.0]), and Google Scholar (88.5 [31–169.50] vs. 75 [5–152])}. Meanwhile, 11/52 (21.2%) co‐publications achieved higher MEDLINE citations rates than the original review (Figure 2). There was no discernable pattern of these reviews in terms of year published, topic, or journal. Table 1 lists individual Cochrane reviews, irrespective of associated co‐publication, with the highest numbers of MEDLINE citations (irrespective of whether there was a co‐publication or not) and Table 2 details co‐publications with the highest numbers of citations.

**Table 1:** Top 10 urologic Cochrane reviews cited on MEDLINE.

Title	MEDLINE citations	Area	Group	Year
Surgical management of pelvic organ prolapse in women (apical vaginal prolapse)	277	Pelvic organ prolapse	Incontinence group	2016
Surgical management of pelvic organ prolapse in women (anterior prolapse)	247	Pelvic organ prolapse	Incontinence group	2004
Screening for prostate cancer	195	Prostate cancer	Urology group	2006
Cranberries for preventing urinary tract infections	192	Urinary tract infection	Kidney/Transplant group	2003
Pelvic floor muscle training versus no treatment, or inactive control treatments, for urinary incontinence in women	155	Incontinence	Incontinence group	2010
Extracorporeal shock wave lithotripsy (ESWL) versus percutaneous nephrolithotomy (PCNL) or retrograde intrarenal surgery (RIRS) for kidney stones	142	Urinary tract stones	Kidney/Transplant group	2009
Mid‐urethral sling operations for stress urinary incontinence in women	139	Incontinence	Incontinence group	2009
*Serenoa repens* for benign prostatic hyperplasia	87	Benign prostatic hypertrophy	Urology group	2002
Anticholinergic drugs versus placebo for overactive bladder syndrome in adults	84	Incontinence	Incontinence group	2002
Antibiotics for preventing recurrent urinary tract infection in nonpregnant women	84	Urinary tract infection	Kidney/Transplant group	2004

**Table 2:** Top 10 Cochrane co‐publications cited on MEDLINE.

Title	MEDLINE citations	Area	Journal	Year
Adjuvant chemotherapy in invasive bladder cancer: a systematic review and meta‐analysis of individual patient data	126	Bladder cancer	Eur Urol	2005
Targeted therapy for advanced renal cell cancer (RCC): a Cochrane systematic review of published randomized trials	110	Kidney cancer	BJU Int	2011
Palliation of metastatic bone pain: single fraction versus multifraction radiotherapy—a systematic review of randomized trials	62	Bone metastasis	Clin Oncol Res	2002
Systematic review and meta‐analysis of monotherapy compared with combined androgen blockade for patients with advanced prostate carcinoma	60	Prostate cancer	Cancer	2002
A systematic review of intravesical bacillus Calmette‐Guérin plus transurethral resection versus transurethral resection alone in Ta and T1 bladder cancer	56	Bladder cancer	BJU Int	2001
A systematic review and meta‐analysis of randomized trials of neo‐adjuvant hormone therapy for localized and locally advanced prostate carcinoma	43	Prostate cancer	Cancer Treat Rev	2008
Systematic review of the relative efficacy of nonsteroidal anti‐inflammatory drugs and opioids in the treatment of acute renal colic	42	Urinary tract stones	BMJ	2004
Pelvic floor muscle training versus no treatment, or inactive control treatments, for urinary incontinence in women: a short version Cochrane systematic review with meta‐analysis	41	Incontinence	Neurourol Urodyn	2015
Diagnosis and treatment of chronic abacterial prostatitis: a systematic review	34	Prostatitis	Ann Intern Med	2000
Prostate magnetic resonance imaging, with or without magnetic resonance imaging‐targeted biopsy, and systematic biopsy for detecting prostate cancer: a Cochrane systematic review and meta‐analysis	32	Prostate cancer	Eur Urol	2019

*Note*: Ann Intern Med, Annals of Internal Medicine; BJU Int, British Journal of Urology International; BMJ, British Medical Journal; Cancer Treat Rev, Cancer Treatment Reviews; Clin Oncol Res, Clinical Oncology and Research; Eur Urol, European Urology; Neurourol Urodyn, Neurourology and Urodynamics.

## 4 DISCUSSION

We found that approximately one in four urology‐related Cochrane reviews is co‐published. Though a wide spectrum of journals co‐published urologic Cochrane reviews, BJU International was the primary journal for most co‐published reviews. Co‐publications also garnered a considerable number of citations, although usually less than the original reviews. It is important to note that co‐publications may not be readily identifiable as such since only about half included Cochrane in their name. However, this issue appears to be improving as recent co‐publications satisfy this criterion more often.

Our study represents the first formal assessment of Cochrane reviews on urology‐related topics. All aspects of this study were governed by an apriori protocol using a data abstraction form that was pilot‐tested in advance. Data abstraction and citation analysis were performed by one of two members of the investigative team (Ranveer Vasdev and Zahrah Shakur) with independent verification of a random subset of 20% co‐publications for accuracy which found a high level of concordance. Citation analyses were not limited to MEDLINE but also include the Web of Science, Scopus, and Google Scholar.

One important limitation of this study is that we did not control the citation analysis for time‐of‐publication. Therefore, reviews published earlier will have had the opportunity to be cited more often and skew the impact analysis when compared to later studies. Second, we recognize that citation analyses (even when using multiple databases) are only one way to measure impact. Other approaches such as the use of Altimetric scores or assessing whether a given review was quoted in a clinical practice guideline, were not utilized. Last, our analysis was limited to publications written in English.

The most comparable study to ours assessed Cochrane reviews and resulting co‐publications in ophthalmology. [4] The authors found a total of 117 original reviews (published until 2014) of which 19 resulted in 22 corresponding co‐publications. They reported modest adherence to the Cochrane policy on co‐publication with all co‐publications complying with at least one of the four requirements and only half citing the original review. In contrast to our findings, co‐publications commanded approximately 3.5 times the number of citations than the original reviews. Based on these findings, the authors concluded that co‐publication of Cochrane reviews should be encouraged to increase their impact, but that enhanced efforts are needed to make sure they are identifiable as such. We concur with these conclusions.

A more recent study compared random sample of 101 co‐published Cochrane reviews across specialties with a 202 nonco‐published Cochrane reviews matched by year or publication and Editorial Group. [5] They found that Cochrane reviews with co‐publications had more citations than Cochrane reviews without co‐publication, which is a outcome, we intentionally did not analyze in our study since this finding seems expected: Cochrane editorial groups are likely to promote co‐publication of reviews of greatest clinical relevance and newsworthiness, which would also be expected to garner more citations. Based on their citation analysis, they also concluded that citations from co‐publications helped approximately one of four co‐publishing journals increase their impact factor. While not formally assessed, we would assume the same to be true for the co‐publishing journals in our study, most of which have had modest impact factors of 5 or less during the study period. We intentionally did not utilize impact factor as a covariate outcome as this measure is more representative of a specific journal's readership rather than the co‐publication or review of interest.

Previous studies have provided longitudinal analyses of the methodological quality of systematic reviews published in the urological literature, which has historically been poor. [3, 6, 7, 8] These have included small numbers of co‐publications of Cochrane reviews that stand out for their much higher compliance with Assessment of Multiple Systematic Reviews (AMSTAR) [9] and AMSTAR‐2 criteria, [10] for example, with regard to existence of an apriori protocol or explicit reporting of studies excluded at the full‐text stage. [3] Therefore, an added benefit of co‐publication may be to raise awareness for what high‐quality systematic reviews should look like and helping to raise methodological standards over time.

Findings of this study suggest that co‐publications of Cochrane reviews make a substantial contribution to the dissemination of reliable evidence summaries. Since only approximately one in four urologic Cochrane reviews are co‐published, efforts should be made to increase the number of co‐publications and potentially expand the number of urology specialty journals with whom co‐publication agreements are established; this would appear to be of mutual value to both Cochrane as well as the co‐publishing journal. However, it is pertinent that these co‐publications are readily identifiable as such, which means they need to include “Cochrane” in their titles as well as satisfy the four criteria for co‐publication. The responsibility of assuring that is the case should be shared by the authors who are submitting for co‐publication and the editorial groups that approve the co‐publication and oversee compliance with other aspects governing co‐publications, such as consistent authorship.

Whereas we and others [4, 5] believe that co‐publications provide added value by bringing Cochrane reviews to the awareness of clinicians, guideline developers, and health policy decision‐makers who do not search the Cochrane Library and otherwise would not know about these reviews, there is the potential concern that co‐publications draw attention away from, and reduce the citation impact of, the actual reviews. This notion is hard to prove or disprove. The definitive approach to addressing this question would be to randomize a set of similar Cochrane reviews at their time of publication to co‐publication, or not. However, each review is likely unique, and the date of publication would also be a variable that matters and could not be easily controlled for.

One potential future study would be to determine whether urology guidelines citing Cochrane reviews, cite the original documents, the co‐publication, or both. For guideline documents it would appear appropriate to reference the original full review since it would include not only all methodological details but also a full account of what the review found.

## 5 CONCLUSION

Approximately one in four urology‐related Cochrane reviews are co‐published in an abbreviated form in a specialty journal, although not all co‐publications are readily identifiable as such. Co‐publications garnered a considerable number of citations, although usually less than the original reviews. Increased efforts to promote co‐publications in specialty journals, respecting Cochrane criteria for co‐publication, would be helpful for disseminating high‐quality evidence synthesis as well as promoting higher methodological standards for systematic reviews in the urological literature.

## AUTHOR CONTRIBUTIONS

**Ranveer Vasdev**: Conceptualization, data curation, formal analysis, investigation, project administration, validation, writing—original draft, writing—review and editing. **Zahrah Shakur**: Data curation, formal analysis, investigation, validation, writing—review and editing. **Philipp Dahm**: Conceptualization, investigation, methodology, project administration, supervision, writing—original draft, writing—review and editing.

## CONFLICT OF INTEREST STATEMENT

The authors declare no competing interests.

## ETHICS STATEMENT

Institutional Review Board approval was not required as no individual patient data was utilized. The data reported in this manuscript are complete and accurate, and all data were handled in a secure manner.

## Supporting information

Supplementary information.

Supplementary information.

## Data Availability

The data that support the findings of this study are available from the corresponding author upon reasonable request.
