# Supplementary material for: Criteria adherence and citation impact of urologic Cochrane review co‐publications
Source: Cochrane Evid Synth Methods. 2023 Mar 27;1(2):e12004. doi: 10.1002/cesm.12004 (PMC11795977; doi:10.1002/cesm.12004)
Supplement: Supplementary file 1 — Supplementary information. [file CESM-1-e12004-s002.docx]

**Included Topics:** lower urinary tract symptoms, urinary tract infections, cystitis, pyelonephritis, bacteriuria, hydroceles, stones, hypercalciuria, sexual dysfunction, phimosis, circumcision

**Excluded Topics:** acute kidney injury, chronic kidney disease, autosomal dominate polycystic kidney disease, hemolytic uremic syndrome, dialysis, renal vasculitis, nephrotic syndrome, kidney function, or diabetic kidney

**Appendix 1:** Topics Included and Excluded from the Kidney and Transplant Editorial Group
