# Supplementary figures and images for: Criteria adherence and citation impact of urologic Cochrane review co‐publications
Source: Cochrane Evid Synth Methods. 2023 Mar 27;1(2):e12004. doi: 10.1002/cesm.12004 (PMC11795977; doi:10.1002/cesm.12004)

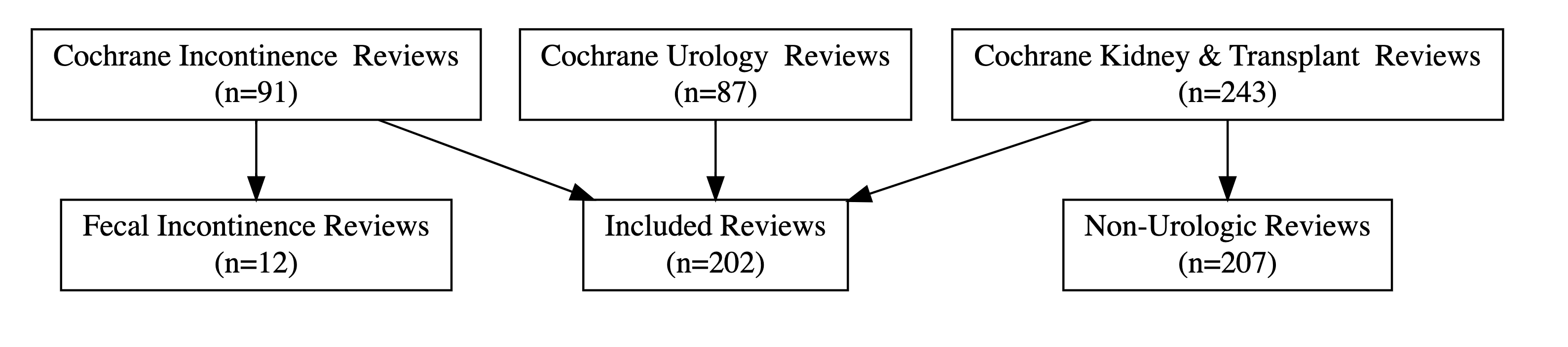


**Appendix 2**: Cochrane Review Flow Diagram

Supplement: Supplementary file 2 — Supplementary information. [file CESM-1-e12004-s001.docx]
